# Pharmacokinetics of Ampicillin Trihydrate in Plasma, Interstitial, and Peritoneal Fluid Following Intraperitoneal or Intramuscular Administration in Steers at the Beginning of a Standing Flank Laparotomy

**DOI:** 10.1111/jvp.70023

**Published:** 2025-09-04

**Authors:** Danielle A. Mzyk, Jennifer L. Halleran, Laura M. Neumann, Ronald E. Baynes, Derek M. Foster

**Affiliations:** ^1^ Department of Population Health and Pathobiology North Carolina State University College of Veterinary Medicine Raleigh North Carolina USA

**Keywords:** ampicillin, cattle, interstitial, intraperitoneal, pharmacokinetics

## Abstract

Prophylactic and perioperative use of antibiotics is common prior to abdominal surgery in cattle for minimizing the risk of postoperative infections. Yet, there is little information on drug concentrations at sites of potential infections following surgical procedures. The objective of this study was to compare the concentrations in the plasma, peritoneal fluid, and interstitial fluid of ampicillin trihydrate in cattle. In a randomized design, ampicillin trihydrate, a β‐lactam antibiotic, was administered to 12 healthy Holstein‐Friesian steers intraoperatively via intraperitoneal (IP; *n* = 6) or intramuscular (IM; *n* = 6) injection in the cervical neck muscles at 11 mg/kg for both groups. For IP administration, ampicillin trihydrate was deposited into the abdominal cavity following an incision in the right paralumbar fossa. Steers in the IM group were administered ampicillin prior to surgical closure. Peritoneal fluid and interstitial fluid were collected using ultrafiltration probes. IP administration achieved higher concentrations in peritoneal fluid as compared to IM administration. Maximum plasma concentrations were significantly higher following IP administration (3.11 ± 2.5 μg/mL; *p* < 0.004) compared to the IM group (0.05 ± 10.9 μg/mL). Despite high peritoneal fluid concentrations of ampicillin, the variability in critical pharmacokinetic parameters following IP administration raises concerns about its therapeutic reliability. The correlation between intraperitoneal drug concentrations and clinical efficacy warrants further investigation.

## 1 Introduction

The prophylactic use of antibiotics is common prior to abdominal surgery in cattle as many surgical procedures are routinely performed by cattle veterinarians on farms (Chicoine et al. 2008). The rationale behind this practice is to reduce the microbial counts at the surgical site, thereby minimizing the risk of postoperative infection. Antibiotic prophylaxis is typically given before contamination occurs, often within 60 min before surgical incision. Drugs administered perioperatively (defined as around the time of surgery) can be classified as preoperative (before surgery), intraoperative (during surgery), and postoperative (after surgery). Cattle receiving preoperative prophylaxis have demonstrated significantly greater postoperative feed intake, lower rectal temperatures, and fewer abscesses at the surgical site than those receiving no antibiotics (Haven et al. 1992). Perioperative antibiotics may serve both prophylactic and therapeutic roles, depending on timing, infection risk, and patient factors.

With the growing concern of antimicrobial resistance, the selection of an appropriate therapy, including the route, dosage, timing, and duration of the treatment, is of high importance.

Ampicillin (AMP), a beta‐lactam antibiotic, has bactericidal activity against a wide range of common gram‐positive and gram‐negative bacteria which can contribute to surgical site infections. AMP is approved for use in cattle for the treatment of pneumonia. Although AMP has a similar mechanism of action to other penicillin antibiotics used in cattle, ampicillin has an additional amino group which helps it penetrate the outer membrane of gram‐negative bacteria. The limited effectiveness of ampicillin against Enterobacterales represents a significant gap in antimicrobial coverage, particularly given the potential involvement of organisms like *Escherichia coli* in surgical site infections. The antibacterial action of ampicillin depends on reaching the minimal inhibitory concentration (MIC) for a certain percentage of the dosing period. When changing the route or method of administration, it is important to investigate how this affects drug concentration at the site of infection to observe if target attainment against suspected pathogens will be reached. The major benefit of intraperitoneal (IP) administration is the relatively high concentrations directly within the peritoneum. There was greater availability of cephalosporins in peritoneal fluid following IP administration when compared to the intravenous route in horses (Alonso et al. 2018, 2021). Intraoperative IP administration of procaine G penicillin showed rapid absorption and elimination in cattle, which reached higher plasma concentrations more quickly than intramuscular administration (Chicoine et al. 2009).

Formulation differences of ampicillin have been studied in cattle following parenteral administration, and there is considerable variability in calculated pharmacokinetic parameters among published studies (Klein et al. 1989; Gehring et al. 2005). It cannot be assumed that additional ampicillin formulations produce similar results and, therefore, should be evaluated. The aim of this study was to compare the profiles of plasma and peritoneal fluid concentrations of ampicillin trihydrate after IP and intramuscular (IM) administration in steers undergoing exploratory abdominal surgery. A secondary objective was to evaluate the distribution of unbound ampicillin within the peritoneal cavity, surgical site, and subcutaneous fluid and to evaluate the possible adverse effects of IP administration. We hypothesized that ampicillin trihydrate administered IP to cattle would produce high concentrations of ampicillin in plasma and peritoneal fluid but would be rapidly eliminated, while IM administration would produce sustained drug concentrations in the peritoneal fluid that would be more likely to reach therapeutic targets in cattle undergoing abdominal surgery.

## 2 Materials and Methods

### 2.1 Animals

A total of 12 healthy 5‐month old Holstein/Jersey cross steers were purchased from a local dairy farm. The mean weights of all steers ± standard deviation were 180.9 ± 40.1 kg (range 111–256.5 kg). Upon arrival, steers were examined by a veterinarian and deemed healthy for enrollment based upon physical examination. No steers used in the study had undergone prior abdominal surgery and they had not received any recent antimicrobial therapy prior to enrollment. Steers were individually housed in stalls at the Lab Animal Resources Facility at North Carolina State University College of Veterinary Medicine for the duration of the trial and were fed a diet of alfalfa/grass hay and livestock pellets. Water was freely available. The study protocol was approved by Institutional Animal Care and Use Committee (IACUC) of the North Carolina State University (Protocol #23‐372). Using an online random group generator (https://static.sps.ed.ac.uk/groupallocator/), each steer was randomly allotted to a group receiving ampicillin trihydrate (11 mg/kg) by either (1) single dose, intraoperative intraperitoneal administration or (2) single dose, intraoperative intramuscular administration. 11 mg/kg was selected for the dose for both IP and IM administration based on the approved product dosing for cattle in the United States. Each steer was administered a single intravenous dose of flunixin meglumine (2.2 mg/kg; Prevail [50 mg/mL]; VetOne, Boise, ID) immediately following the surgical procedure and 24 h after surgery either received a 2nd dose of IV flunixin or a single dose of transdermal flunxin meglumine solution (3.3 mg/kg; Banamine Transdermal [50 mg/mL]; Merck Animal Health, Rahway, NJ). As part of a different study, each steer was pain scored by a board certified expert in anesthesia and analgesia and was administered rescue analgesia (morphine sulfate at 0.1 mg/kg IM) if needed. Only one steer was determined to need rescue analgesia post‐surgery. All steers were humanely euthanized at the end of the study period following sedation with xylazine (0.1 mg/kg; XylaMedTM Injection [100 mg/mL]; VetOne, Boise, ID) with intravenous administration of pentobarbital/phenytoin (Euthasol 390 mg/mL; Verbac, Westlake, TX).

### 2.2 Placement of Ultrafiltration Probes

**FIGURE 1:**
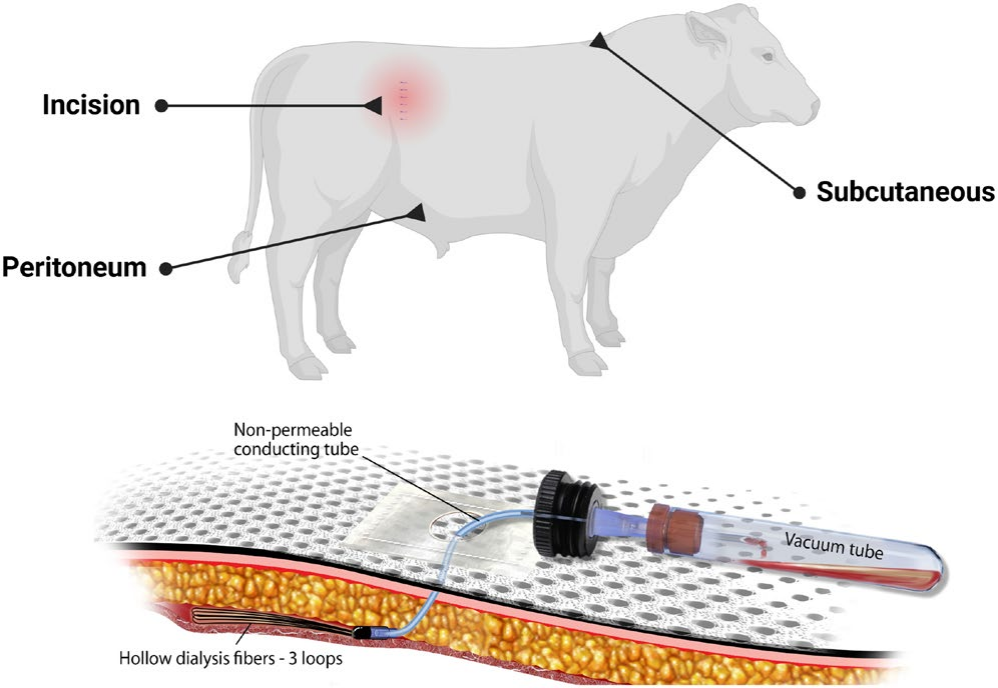
Ultrafiltration probe locations for sampling in steers.

The steers were fitted with multiple in vivo ultrafiltration sampling kits (BAS Bioanalytical Systems, W. Lafayette, IN) as previously described (Warren et al. 2014; Foster, Jacob, et al. 2016; Foster, Martin, and Papich 2016; Mzyk et al. 2019) in three locations (Figure 1): subcutaneous space caudal to the withers on the dorsal midline; at the site of the surgical incision and within the peritoneal cavity. Ultrafiltration probes were placed subcutaneously over the withers prior to surgery to collect interstitial fluid for 24 h, while the probes at the site of the incision and within the peritoneum were placed at the time of surgical closure. Each probe consists of three semipermeable loops linked to a non‐permeable tube that extends outside the animal and connects to an evacuated blood collection tube without additives. The evacuated tube generates negative pressure, facilitating fluid collection through the small pores in the loop membrane. This membrane contains pores that permit the passage of water, electrolytes, and low molecular weight molecules (under 30,000 Da) while preventing the transfer of proteins, protein‐bound drugs, and other large molecular weight compounds. During the surgical procedure (described below), an ultrafiltration probe was placed in the peritoneal cavity. After entering the abdomen, the surgeon pushed an introducer needle through the right caudoventral abdominal wall from the abdominal cavity through the skin. An assistant aseptically fed an ultrafiltration probe through the needle to the surgeon. The surgeon gently held the tubing of the probe in place in the abdomen while the assistant removed the needle and secured the probe to the skin. The ultrafiltrate fluid was collected at Time 0, 2, 4, 6, 8, 10, 12, 18, and 24, 30, 36, 42, and 48 h after administration of ampicillin trihydrate. The fluid was collected and immediately frozen at −80°C and stored until analysis. These samples were stored for 18 months prior to analysis.

### 2.3 Jugular Catheter Placement and Plasma Sample Collection

Prior to surgery, a 14 gauge, 13 cm jugular catheter (Milacath; MILA, Hebon, KY) was aseptically inserted in the jugular vein and an extension set was then attached and both were sutured in place using a 2–0 monofilament suture. Blood samples were collected at time 0, and at 15, 30, 60, 120 min, and 4, 6, 8, 10, 12, 18, and 24 h after initial ampicillin trihydrate administration.

### 2.4 Surgical Procedure and Drug Administration

Intraperitoneal ampicillin administration was designed to mimic a typical dosing time performed during a routine bovine abdominal surgery. The right paravertebral fossa was clipped and aseptically prepared for surgery. To provide local anesthesia of the right flank, a distal paravertebral block with lidocaine was performed, using a total volume of 20 mL spread proximal and distal to the transverse processes of L1, L2, and L4. A vertical 20‐cm incision was made 6 cm below the transverse processes of the lumbar vertebrae, approximately equidistant from the last rib to the tuber coxae in the paralumbar fossa. Sharp dissection continued through the external and internal abdominal oblique muscles, transverse abdominal muscle, and peritoneum to enter the peritoneal cavity. The cecum was identified and exteriorized caudally to expose the ileocecal fold. The ileocecal fold was used to identify the ileum, and ultrafiltration probes were placed in the ileum and colon as previously described as part of another study (Warren et al. 2014). Prior to closure of the abdomen, ampicillin trihydrate (11 mg/kg) for the IP group or an equivalent volume of sterile saline in the IM group was infused into the abdominal cavity between the greater omentum and abdominal wall. For the IP group, an equivalent volume of saline to the ampicillin dose was injected IM in the cervical neck area. For steers in the IM group, a single dose of ampicillin trihydrate was administered IM in the cervical neck muscles at an equivalent time in the surgical procedure. If the volume to be injected was > 10 mL, the dose was split into two equal volumes per injection site on the same side of the neck. After administration of ampicillin trihydrate or saline into the abdomen, the peritoneum, muscle, and skin layers were closed with routine surgical methods in a three‐layer closure. The internal abdominal oblique and transversus muscle layers were closed together, using 1 vicryl suture in a simple continuous pattern. The external abdominal oblique layer was closed by itself in a similar fashion. The skin layer was closed using 1# braunamid suture in a ford interlocking pattern.

### 2.5 Drug Concentration Analysis

#### 2.5.1 Solvents and Reagents

Ampicillin trihydrate was purchased from Millipore Sigma (Burlington, MA, USA). LC–MS grade acetonitrile, methanol, 2‐propanol, and formic acid were purchased from Fisher Scientific (Thermo Fisher, Waltham, MA, USA). Ultrapure water was obtained in‐house from a Millipore Synergy UV water purification system (Millipore Sigma).

#### 2.5.2 Sample Preparation for Bovine Plasma

The plasma was centrifuged at 10,000 × *g* for 5 min. One hundred microliter of the supernatant was mixed with 400 μL of ultrapure water in a microcentrifuge tube. A Waters Oasis Prime HLB μElution plate (Waters Corporation, Milford, MA, USA) was conditioned with 500 μL of methanol followed by 500 μL of ultrapure water using a Waters Otto SPEcialist (Waters Corporation). The diluted plasma was loaded into the μElution plate, then washed with 500 μL of 95:5 water:methanol. A collection well plate was placed under the μElution plate, using the proper spacers, for sample elution with 100 μL of 90:10 acetonitrile:methanol. The collection well plate was covered with a cap mat, then placed into the sample manager for analysis by ultra‐performance liquid chromatography/mass spectrometry (UPLC/MS/MS). The calibration ranges of 0.005–1 μg/mL were linear with a coefficient of determination, *R* ^2^, > 0.99. Each calibration standard concentration could be back calculated to within 15% of the true concentration. The intra‐day precision and accuracy for plasma were calculated and can be found in Table S1. Inter‐day precision and accuracy for plasma are shown in Table S2. The limit of detection and limit of quantification were recognized as 0.001 and 0.005 μg/mL in plasma.

#### 2.5.3 Sample Preparation for Bovine Interstitial Fluid (ISF)

Blank ISF from previous bovine studies was used for making the standard curve for both peritoneal and ISF samples. Twenty microliter of control blank bovine ISF samples were spiked with 40 μL of the appropriate ampicillin concentrations in ultrapure water to prepare the matrix‐matched calibration plot. Forty microliter of ultrapure water only was added to 20 μL of the other samples to give a total volume of 60 μL in all the ISF samples. All samples were vortex mixed briefly, then filtered through 0.2‐μm pore size PVDF syringe filters into 0.6‐mL microcentrifuge tubes. Note that for the non‐spiked samples, 40 μL of the ultrapure water was added to all the tubes first before adding the 20 μL of sample. The samples were transferred into a Waters Acquity UPLC 700 μL round 96‐well sample plate (Waters Corporation) then covered with a cap mat for analysis by UPLC/MS/MS.

The calibration ranges of 0.001–1 μg/mL were linear with a coefficient of determination, *R* ^2^, > 0.99. The intra‐day precision and accuracy for ISF were calculated and can be found in Table S3. Inter‐day precision and accuracy for ISF are shown in Table S4. The limit of detection and limit of quantification were recognized as 0.0005 and 0.001 μg/mL.

#### 2.5.4 UPLC/MS Conditions for Bovine Plasma

A Waters Acquity Ultra Performance Liquid Chromatograph coupled to a Waters Acquity QDa mass spectrometer (Waters Corporation, Milford, MA, USA) was programmed for single ion recording (SIR) of 350.1175 m/z using electrospray ionization in the positive mode (ES+). The cone and capillary voltages were 10 and 0.8 V, respectively. The separations were performed on a Waters Acquity UPLC BEH C8 1.7 μm (2.1 mm × 100 mm) column with the corresponding VanGuard pre‐column (Waters Corporation). The mobile phase was programmed for a gradient where solvent A1 was 0.1% formic acid in water and solvent B1 was 0.1% formic acid in acetonitrile. Initially, A1:B1 was 90:10 for 1 min. Between 1 and 2.50 min, A1:B1 changed linearly to 10:90, where it was then held until 3.50 min. At 3.51 min, A1:B1 was returned to its initial condition of 90:10 and held until 5.00 min. The total run time was 5 min. The column and sample temperatures were 35°C and 20°C, respectively. The injection volume was 5.0 μL. The weak needle wash composition was 90:10 water:acetonitrile. The strong wash was 1:1:1:1 water:acetonitrile:methanol:2‐propanol. The strong wash volume was 200 μL. The weak wash volume was 600 μL.

#### 2.5.5 UPLC/MS/MS Conditions for Bovine Interstitial Fluid (ISF) or Ultrafiltrate (UF)

Sample analysis of ISF was performed on a Waters Acquity I‐Class Ultra Performance liquid chromatograph coupled to a Waters Xevo TQD tandem mass spectrometer (Waters Corporation, Milford, MA, USA). The column, gradient mobile phase conditions, run time, column and sample temperatures, and injection volume were the same as for the cow plasma analysis described previously. The Xevo TQD was programmed for MRM of two mass pairs. The parent m/z was 350.07 for both mass pairs. The daughters were 105.95 m/z and 160.01 m/z. For quantitation, the 350.07 > 105.95 m/z trace with a cone voltage of 28 V and collision energy of 16 V was used. The ionization mode was ES+.

### 2.6 Pharmacokinetic Analysis

Individual curves of ampicillin plasma and peritoneal fluid concentration versus time were constructed for each animal. The plasma concentrations from one steer from each group (IM and IP administration) were excluded from analysis due to only having three plasma samples with quantifiable ampicillin concentrations. The plasma drug concentrations were analyzed using standard pharmacokinetic methods to determine the drug disposition for each drug in each steer. A computer program (Phoenix, V. 8.3; Pharsight Corporation, Certara, St. Louis, MO, USA) was used to determine pharmacokinetic (PK) parameters. The maximum concentration (*C*_max_) and the time of occurrence of the maximum concentration (*T*_max_) for all sample types were obtained from individual observed values. Specific models (one, two, etc., compartments) were determined for best fit on the basis of visual analysis for goodness of fit and by visual inspection of residual plots. A one compartment model with uniform weighting and lag time was selected as the best model for all matrices. Plasma, interstitial fluid, and peritoneal fluid concentrations were based on the equation described in the following formula:

$$C=\frac{k_{01}\mathrm{FD}}{V\;\left(k_{01}-k_{10}\right)}\;\left(e^{-k_{10}t}-e^{-k_{01}t}\right)$$

where *C* is the plasma concentration, *t* is time, *k*_01_ is the extravascular absorption rate (assuming first‐order absorption), *k*_10_ is the elimination rate constant, *V* is the apparent volume of distribution, *F* is the fraction of drug absorbed, and *D* is the extravascular dose. Secondary parameters calculated from the model included the peak concentration (*C*_max_), time to peak concentration (*T*_max_), area under the plasma concentration versus time profile (AUC), absorption and terminal half‐lives (*t*_1/2_). The mean residence time (MRT) was determined using non‐compartmental analysis for each steer with available data. All PK parameters are reported as geometric mean ± geometric standard deviation except for absorption half‐life (*K*_01_ *T*_1/2_) and terminal half‐life (*K*_01_ *T*_1/2_) which are reported as a harmonic mean ± harmonic standard deviation.

### 2.7 Statistical Analysis

Drug concentrations in all matrices are expressed as arithmetic mean and SD. Statistical analysis was performed using GraphPad Prism version 5.00. Pharmacokinetic parameters were compared between the IM and IP groups in plasma, peritoneal fluid, and interstitial fluid (from the subcutaneous probe) using a Mann–Whitney test. Statistical significance was defined as *p* < 0.05. Following IM administration, there were too few sample numbers with quantifiable ampicillin concentrations in interstitial fluid at the site of the incision, so no statistical analysis was performed comparing these groups.

## 3 Results

### 3.1 Pharmacokinetic Analysis

In all figures and tables, the plasma concentrations are reported as the total concentrations (protein bound and free), whereas the concentrations shown in the interstitial fluid collected from the subcutaneous, peritoneal, and incision site probes are only the free drug (microbiologically active) concentrations.

#### 3.1.1 Plasma

**FIGURE 2:**
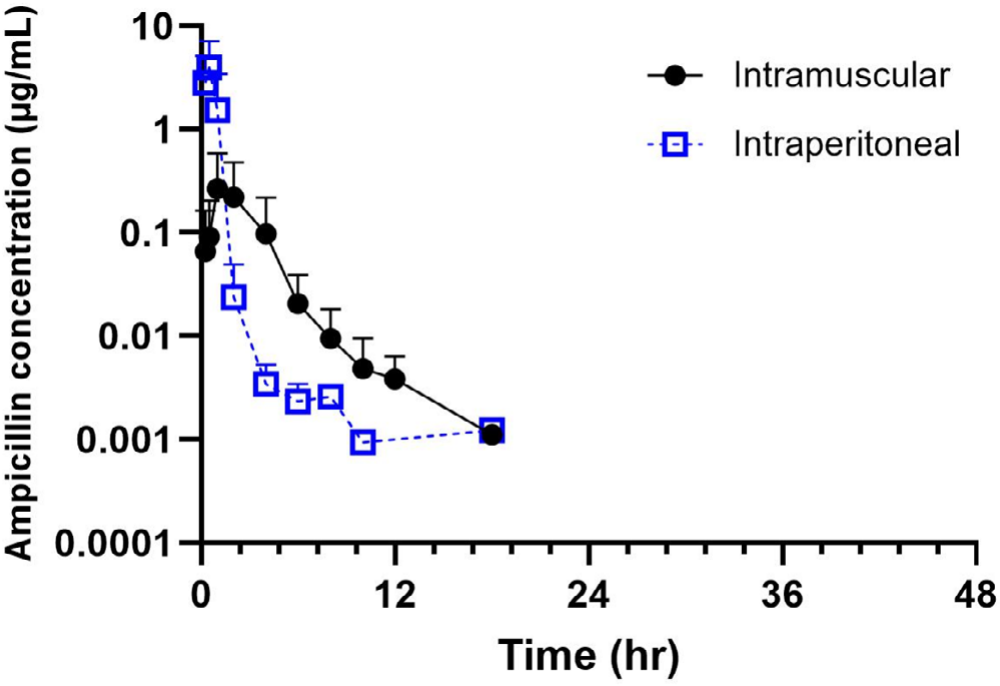
Mean plasma concentration–time curves and standard deviations of ampicillin following intraperitoneal (IP, 11 mg/kg; *n* = 5), intramuscular (IM, 11 mg/kg; *n* = 5) administrations in steers.

No adverse effects were observed immediately after IP or IM administration in any steer. Plasma samples from one steer in each group were removed due to concentrations being below the limit of detection and were excluded from analysis. The mean plasma ampicillin concentration versus time graph for both routes of administration is shown in Figure 2. Following IM injection, the mean maximum plasma concentration (0.05 ± 10.9 μg/mL) was significantly lower (*p* < 0.004) than the plasma concentration reached following IP administration (3.11 ± 2.5 μg/mL). The time to maximum concentration was shorter following IP (0.40 ± 1.43 h) as compared to the IM group (1.26 ± 1.76 h). Mean residence time was significantly longer in calves administered via the IM route (3.61 ± 1.59 h) compared to IP (0.79 ± 1.68 h). Terminal *T*_1/2_ for ampicillin in plasma was 1.08 and 0.51 h for the IM and IP groups, respectively (Table 1).

**TABLE 1:** Plasma ampicillin pharmacokinetics after administration of 11 mg/kg ampicillin trihydrate to steers fit to a one‐compartmental model.

Parameter	Units	Intramuscular			Intraperitoneal			*p*
		*n* = 5			*n* = 5			
		Mean	SD	Range	Mean	SD	Range	
*k*_01_	1/h	0.7	1.3	0.5–1.1	1.9	2.6	0.8–8.0	0.05
*k*_10_	1/h	0.4	2.7	0.1–1.4	1.0	2.4	0.4–2.8	0.16
AUC	h × μg/mL	0.2	6.2	0.04–2.5	0.2	17.4	0.01–2.4	0.99
*k*_01_ *T*_1/2_	h	0.9	0.2	0.6–1.4	0.3	3.7	0.09–0.86	0.05
*k*_10_ *T*_1/2_	h	1.1	0.7	0.5–5.5	0.5	1.3	0.25–1.7	0.14
*T*_max_	h	1.2	1.8	1.0–4.0	0.4	1.4	0.25–0.5	0.002
*C*_max_	μg/mL	0.05	10.9	0.003–0.8	3.1	2.5	0.8–7.8	0.004
MRT	hr	3.6	1.6	2.3–6.7	0.8	1.7	0.4–1.7	0.008

*Note:* Results presented as geometric mean ± geometric standard deviation (SD) except for *k*_01_ *T*_1/2_ and *k*_10_ *T*_1/2_, which are reported as harmonic mean ± harmonic SD.

Abbreviations: *C*_max_, peak concentration; *k*_01_, rate constant for absorption; *k*_01_ *T*_1/2_, absorption half‐life; *k*_10_, rate constant for elimination; *k*_10_ *T*_1/2_, elimination half‐life; *T*_max_, time to peak concentration.

#### 3.1.2 Peritoneal Fluid

**FIGURE 3:**
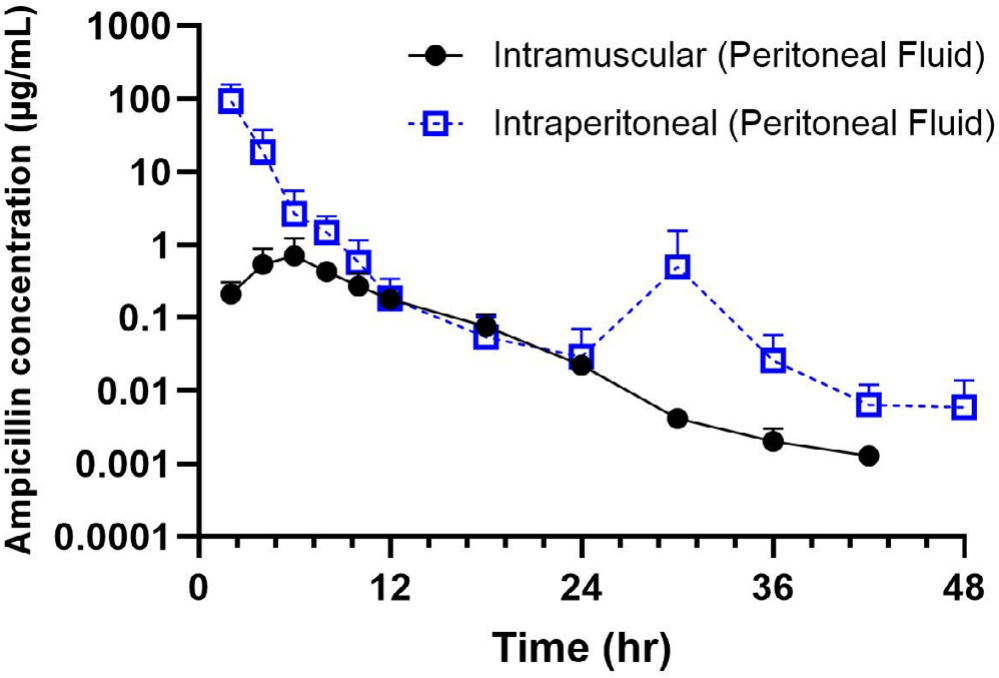
Mean peritoneal concentration–time curves and standard deviations of ampicillin following intraperitoneal (IP, 11 mg/kg; *n* = 6), intramuscular (IM, 11 mg/kg; *n* = 3) administrations in steers.

The comparisons of pharmacokinetic parameters of ampicillin in peritoneal fluid between the routes of administration showed that IP administration provided greater exposure (AUC) of ampicillin than calves administered IM (Table 2). Peritoneal fluid concentrations were significantly higher (*p* < 0.05) following IP administration at 2, 4, 30, and 36 h (Figure 3). The time to maximum concentration was longer following IP administration (5.77 ± 1.42 h) as compared to the IM group (2.3 ± 1.36 h), although not statistically significant. Maximum concentrations determined in peritoneal fluid were higher following IP administration (73.8 ± 1.73 μg/mL) as compared to IM (0.68 ± 1.82 μg/mL). Mean residence time was increased in calves administered ampicillin following the IM route (8.49 ± 1.11 h) as compared to IP (2.88 ± 1.29 h).

**TABLE 2:** Pharmacokinetics of ampicillin in peritoneal fluid after administration of 11 mg/kg ampicillin trihydrate to steers fit to a one‐compartmental model.

Parameter	Units	Intramuscular			Intraperitoneal			*p*
		*n* = 3			*n* = 5			
		Mean	SD	Range	Mean	SD	Range	
*k*_01_	1/h	0.4	2.2	0.2–1.1	4.4	1.4	2.5–5.7	NA
*k*_10_	1/h	0.2	1.4	0.2–0.4	0.8	1.7	0.5–1.9	NA
AUC	h × μg/mL	5.2	1.6	3.6–8.6	371.7	1.7	194.8–801.8	NA
*k*_01_ *T*_1/2_	h	1.2	0.6	0.6–2.8	0.1	1.8	0.1–0.3	NA
*k*_10_ *T*_1/2_	h	2.6	0.1	1.9–3.8	0.7	0.7	0.4–1.4	NA
*T*_max_	h	5.8	1.4	4.0–8.0	2.3	1.3	2.0–4.0	NA
*C*_max_	μg/mL	0.6	1.8	0.4–1.3	73.8	1.7	50.5–183.1	NA
MRT	h	8.4	1.1	7.8–9.6	2.8	1.2	2.2–4.1	NA
AUC_PF_/AUC_plasma_		10.9	4.9	1.9–89.3	1646.9	14.1	112.2–80,176.0	NA

*Note:* Abbreviations listed in Table 1. Results presented as geometric mean ± geometric standard deviation (SD) except for *k*_01_ *T*_1/2_ and *k*_10_ *T*_1/2_, which are reported as harmonic mean ± harmonic SD.

Abbreviations: AUC_PF_/AUC_plasma_, ratio of AUC values of peritoneal fluid:plasma; NA, no statistical analysis performed.

#### 3.1.3 Interstitial Fluid

**FIGURE 4:**
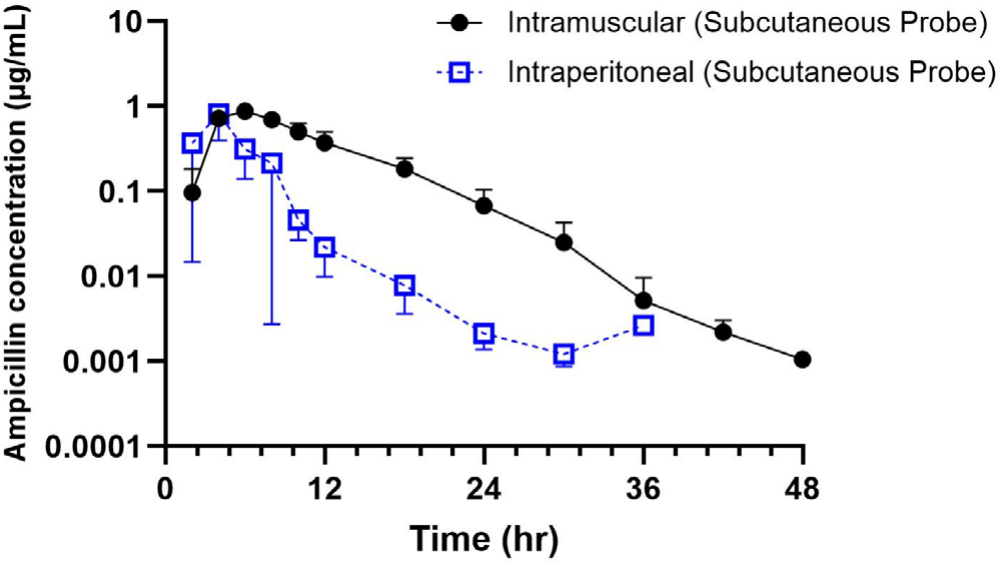
Mean interstitial fluid from subcutaneous site concentration–time curves and standard deviations of ampicillin following intraperitoneal (IP, 11 mg/kg; *n* = 5), intramuscular (IM, 11 mg/kg; *n* = 5) administrations in steers.

**FIGURE 5:**
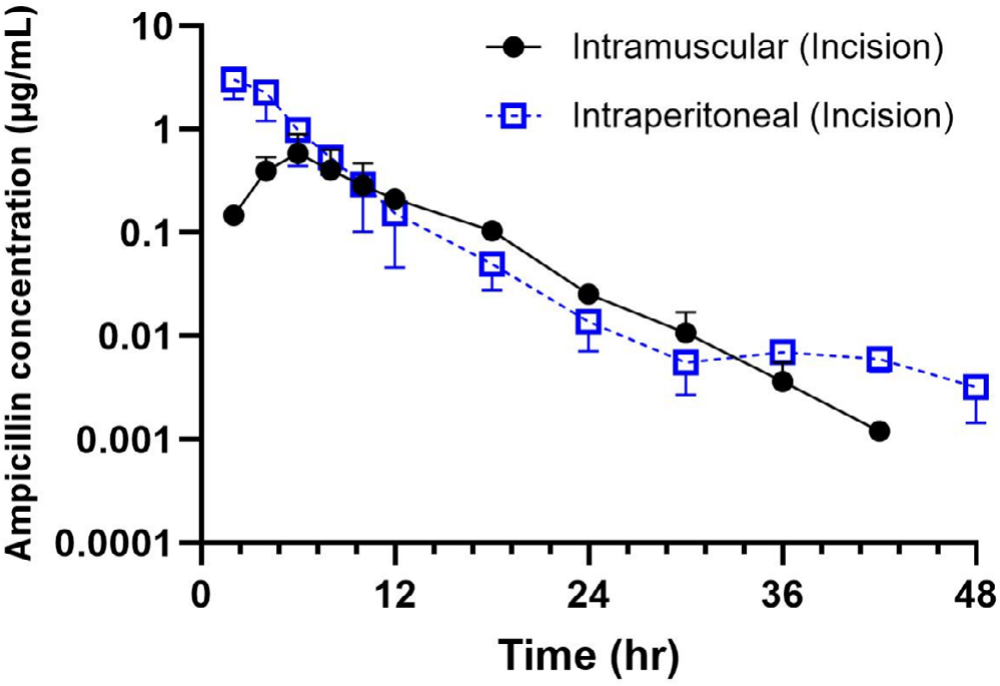
Mean interstitial fluid from the incisional site concentration–time curves and standard deviations of ampicillin following intraperitoneal (IP, 11 mg/kg; *n* = 5), intramuscular (IM, 11 mg/kg; *n* = 2) administrations in steers.

Only two steers had enough data points for the analysis of interstitial fluid from the incisional site probe following IM administration, either from a lack of sample volume collected or from the steers removing the probes before enough samples could be collected for analysis. The pharmacokinetic parameters for interstitial fluid from the subcutaneous and incisional site probes are shown in Tables 3 and 4, respectively. The mean (±SD) concentrations of ampicillin in the ISF collected from the subcutaneous (Figure 4) and incision site (Figure 5) probes are shown following IP and IM administration. The maximum concentration in ISF collected from the subcutaneous probe following IM and IP was 0.82 ± 1.1 μg/mL and 0.69 ± 1.7 μg/mL, respectively.

**TABLE 3:** Pharmacokinetics of ampicillin in interstitial fluid (from subcutaneous probes) concentrations after IP and IM administration of ampicillin trihydrate fit to a one‐compartmental model.

Parameter	Units	Intramuscular			Intraperitoneal			*p*
		*n* = 5			*n* = 5			
		Mean	SD	Range	Mean	SD	Range	
*k*_01_	1/h	0.4	1.6	0.2–0.8	2.7	1.7	1.8–6.5	0.008
*k*_10_	1/h	0.2	1.5	0.1–0.3	0.4	1.9	0.4–1.3	0.04
AUC	h × μg/mL	8.6	1.2	6.7–11.1	2.9	1.5	1.6–3.8	0.008
*k*_01_ *T*_1/2_	h	1.4	0.8	0.8–2.9	0.2	0.1	0.1–0.4	0.008
*k*_10_ *T*_1/2_	h	3.4	1.7	2.7–6.2	1.2	1.0	0.5–2.9	0.03
*T*_max_	h	5.4	1.3	4.0–8.0	3.0	1.5	2.0–4.0	0.07
*C*_max_	μg/mL	0.8	1.1	0.7–0.9	0.6	1.7	0.3–1.3	0.69
MRT	h	10.2	1.1	8.8–12.7	4.3	1.3	2.4–5.7	0.008
AUC_ISF‐SC_/AUC_plasma_		26.3	5.6	3.5–278.0	12.9	9.7	1.5–270.0	0.42

*Note:* Abbreviations listed in Table 1.

Abbreviation: AUC_ISF‐SC_/AUC_plasma_, ratio of AUC values of insterstitial fluid from subcutaneous probe:plasma.

**TABLE 4:** Pharmacokinetics of ampicillin in interstitial fluid from incisional probes concentrations after IP and IM administration of ampicillin trihydrate fit to a one‐compartmental model.

Parameter	Units	Intramuscular			Intraperitoneal			*p*
		*n* = 2			*n* = 5			
		Mean	SD	Range	Mean	SD	Range	
*k*_01_	1/h	0.3	1.6	0.27–0.54	2.8	1.9	1.0–5.5	NA
*k*_10_	1/h	0.2	1.6	0.1–0.3	0.4	1.4	0.2–0.5	NA
AUC	h × μg/mL	4.9	1.5	3.7–6.7	13.6	1.3	10.2–19.2	NA
*k*_01_ *T*_1/2_	h	1.7	0.5	1.3–2.6	0.2	1.8	0.1–0.7	NA
*k*_10_ *T*_1/2_	h	3.3	1.0	2.5–4.8	1.6	1.4	1.2–2.9	NA
*T*_max_	h	6.0	1.0	6.0	2.3	1.4	2.0–4.0	NA
*C*_max_	μg/mL	0.5	1.7	0.3–0.8	3.0	1.4	1.7–3.9	NA
MRT	h	8.8	1.1	7.4–9.8	5.1	1.2	4.0–5.7	NA
AUC_ISF‐INC_/AUC_plasma_		7.9	3.7	2.8–3.4	63.5	11.5	6.4–1701.0	NA

*Note:* Abbreviations listed in Table 1.

Abbreviations: AUC_ISF‐INC_/AUC_plasma_, ratio of AUC values of insterstitial fluid from incisional probe:plasma; NA, no statistical analysis performed.

## 4 Discussion

In humans and veterinary species, the goal of administering preoperative systemic prophylactic antibiotics is to have the concentration in the tissues at its highest at the start and during surgery (Tarchini et al. 2017; Mehta 2020). In large animal medicine, although antibiotic administration intraoperatively is not the optimal time, intraoperative use of antimicrobials offers an improvement over the delayed timing of postoperative administration. Quantifiable ampicillin concentrations were found in the plasma of all steers within 15 min following the administration of ampicillin trihydrate by either route. The absorption and elimination of ampicillin were rapid after IP and IM administration in all steers. The geometric mean peak plasma concentrations obtained in the present study following IM administration (0.05 μg/mL) were lower than those previously reported after IM administration in lactating dairy cattle (1.6 μg/mL following 11 mg/kg) and in calves (3.7 μg/mL following 7.7 mg/kg) (Credille et al. 2015; Nouws et al. 1982). Following IM administration, ampicillin distributes rapidly in the fascial planes or connective tissue surrounding the site of injection. The mean plasma concentrations were much lower in steers from this study as compared to previously published literature. Although an exact reason was not identified, possible explanations include differences in breed and body composition of enrolled animals, perfusion to the site of injection, and differences in volume of injection at each site (Martinez et al. 2001). The average time to maximum plasma concentration (*T*_max_) of 0.4 h was shorter following IP administration as compared to IM administration (1.2 h), and both routes reached peak plasma concentrations sooner when compared to values previously reported in the literature for cattle, although these varied from 0.5 to 8 h (Credille et al. 2015). The early *T*_max_ after IP administration may also be due to the large surface area within the abdomen/peritoneal surface available for drug absorption, as compared to an IM injection.

After absorption from the site of administration, ampicillin is widely distributed in extracellular body fluids. By measuring ampicillin concentrations in the extracellular fluid, this may better correlate pharmacokinetic–pharmacodynamic targets to clinical efficacy (Andes and Craig 2002). Traditionally, pharmacokinetic–pharmacodynamic studies of antimicrobials have regarded plasma drug concentration as a key indicator of potential efficacy. Although plasma drug concentration plays a crucial role in determining the drug's ability to reach the site of infection, the relationship between drug concentration and time at the site of infection may differ significantly from that in the plasma (Liu et al. 2002). In the present study, mean *C*_max_ of ampicillin was significantly greater in peritoneal fluid (73.8 μg/mL) than in plasma (3.11 μg/mL) following IP administration. Concentrations of ampicillin in peritoneal fluid were detected out to 36 h in 3 out of 5 steers (IM administration) and 5 out of 6 steers at 48 h (IP administration). Two out of 5 steers administered IM ampicillin had detectable levels in interstitial fluid collected from the subcutaneous probes at 48 h post dosing. At 24 h, only one steer had detectable levels above the limit of detection in plasma for both routes of administration, similar to previous studies evaluating ampicillin trihydrate (Nouws et al. 1982).

The plasma concentrations from one steer in the IM and IP administration group were excluded from analysis due to only having three plasma samples with quantifiable ampicillin concentrations. Possible explanations for this include an incomplete bioavailability of ampicillin following IM administration. Even though ampicillin trihydrate was administered as an aqueous solution in this study, there is the potential to precipitate once it is injected into the muscle (Martinez et al. 2001). Unabsorbed drug may also remain permanently bound to the muscle tissue and may not be detected in plasma concentrations that fall within the limit of detection.

The peritoneum is made up of a single layer of mesothelial cells and underlying connective tissue that separates the fluid in the cavity from the underlying tissue space but does not provide a significant barrier to transport of molecules (Flessner 2005). In humans receiving chemotherapeutics, the peritoneal transfer of certain drugs may improve due to larger contact surface area as well as uptake by the lymphatics system.

Passive sampling of the ISF allows for the measurement of sequential concentrations in the subcutaneous and peritoneal cavity and over the collection period. Unlike plasma, a time delay of the ISF sampling occurs because of the continuous vacuum of the sampling tube, leading to higher accumulation at each time point between samples. This can lead to accumulation of ampicillin concentrations at the site of sampling, which could influence pharmacodynamic calculations.

In cattle, the main goal of IP administration of antimicrobials during surgery is to reach concentrations above the MIC at the potential infection site, which can be either the peritoneum or the incisional site. β‐lactams are time‐dependent antimicrobials whose efficacy at these sites is mainly related to fT > MIC, which is the amount of time that the unbound drug concentration remains above the MIC of the infecting organism (Drusano 2004). Ideally, unbound drug concentrations at sites of infection should remain above the MIC for 50% of the dosing interval (Drusano 2004). If the infection is at or around the surgical site (including skin, muscle and possibly the peritoneum), prolonged concentrations at these sites would be therapeutically advantageous. Pharmacodynamics describes the antimicrobial effects on the pathogen and involves a complex relationship between antibiotic exposure, microbiological response, and clinical outcomes. This relationship is integral to optimizing antibiotic dosing for the treatment of infections, but there is less research on what is clinically effective for preventing infections post‐surgery in cattle. Clinical endpoints that are required for effective prophylaxis might differ pharmacodynamically from the treatment of active infections. As expected, ampicillin concentrations in peritoneal fluid following IP administration demonstrated an increased area under the concentration (AUC) time curve (371.7 ± 1.7 h × μg/mL) as compared to steers administered by IM route (5.2 ± 1.5 h × μg/mL). In steers that were administered IM ampicillin, the AUC was significantly higher in interstitial fluid collected from the subcutaneous probes (8.6 h × μg/mL) as compared to IP administration (2.9 h × μg/mL), suggesting differences in absorption and distribution to the ISF. Only two steers in the IM group maintained the ultrafiltration probe at the incisional site, so the evaluation of this data is limited. However, it is important to evaluate both interstitial fluid and peritoneal fluid concentrations, as interstitial fluid levels may better reflect tissue drug concentrations at the incisional site during infection than plasma levels. When there is inflammation within the peritoneum, peritoneal fluid may be more predictive of therapeutic concentrations. β‐lactams have been evaluated in horses following IP administration and demonstrated higher and prolonged peritoneal fluid concentrations as compared to intravenous injection (Alonso et al. 2018). The pharmacological effect of ampicillin is closely related to the time exceeding the MIC in plasma. Further research is needed to determine if prolonged peritoneal fluid concentrations of ampicillin after IP administration may be therapeutically advantageous in some clinical cases.

Several limitations need to be considered when evaluating the pharmacokinetic parameters following intraperitoneal administration. For one, previous studies have evaluated the PK following IP administration of antibiotics in lactating dairy cattle (Chicoine et al. 2009). Physiologic differences between steers and dairy cattle, including weight, breed, and production status, need to be considered when evaluating pharmacokinetics. The lack of pharmacokinetic data for IP administered antibiotics in lactating dairy cattle makes comparisons across production classes limited.

Intraperitoneal fluid distribution and contact with the peritoneum add another source of variability (Dedrick and Flessner 1997). Diffusion across stagnant peritoneal fluid may be a rate‐limiting step for absorption of therapeutic compounds (Levitt et al. 1989). Although the six steers that were administered ampicillin trihydrate IP during the surgical procedure were standing, it was noted that some animals went down in the chute for a few minutes while closing the surgery site, which could have influenced the surface area of the peritoneum exposed to ampicillin. In addition, all steers had to be walked back to their stalls from the surgery suite, which may have influenced absorption from the peritoneum following IP administration. At necropsy, evidence of mild inflammation was noted in one steer following IP injection, which consisted of diffuse fibrin attached to the serosal surface of the omentum, but histologic evaluation was not performed. This finding may be due to the potential inflammatory nature of the trihydrate formulation or even the surgical procedure itself. The anhydrous form of ampicillin is significantly more soluble than the trihydrate form at biological temperature in humans and beagle dogs (Poole and Bahal 1968), which could point to formulation as a potential cause of the inflammatory response. In pediatric patients with sepsis, a positive correlation has been noted between inflammatory biomarkers and volume of distribution (Shahrami et al. 2022) Therefore, drugs with poor solubility or those altered in their binding by inflammatory mediators can exhibit decreased penetration into peritoneal tissues or fluid. Since formulation likely impacts the absorption and distribution of ampicillin following parenteral administration, additional studies are warranted.

Culture and susceptibility testing of bacteria causing postsurgical infections is not routinely performed in bovine practice, and were not collected in this study, so we were unable to determine if concentrations of ampicillin reached clinically relevant pharmacodynamic end points. Some practitioners argue that IP antimicrobials exert a localized effect in the abdomen, acting directly at the infection site. However, the rapid absorption from the abdominal cavity challenges this theory, especially since the efficacy of β‐lactams relies on concentrations greater than the MIC of a pathogen for part of the dosing interval.

To evaluate the efficacy of IP versus IM dosing of ampicillin, clinical data is needed on bacteria isolated from surgical sites in cattle. One study evaluated the bacteria encountered in the surgical site during elective cesarean section performed in Belgian Blue cows. Although most of the samples showed a negative bacterial culture, 25% of cows sampled during surgery displayed a positive result. The most encountered bacterial species in that study identified *Acinetobacter* sp., *Pseudomonas* sp., likely from environmental contamination (Djebala et al. 2025). In Belgian Blue cattle affected by fibrinous peritonitis following cesarean section, *Trueperella pyogenes* and *E. coli* were the most frequently identified bacteria in peritoneal exudate (Djebala et al. 2020). In Europe, the epidemiological cutoff value describes the MIC above which bacterial isolates have phenotypically detectable acquired resistance mechanisms. These values are used as part of the clinical breakpoint setting process. If using the European Committee on Antimicrobial Susceptibility Testing wild‐type MIC data for *E. coli* of 8 μg/mL, ampicillin concentrations in peritoneal fluid only reached/maintained concentrations above 8 μg/mL following IP administration for 16%–33% of 24 h. No steers reached peritoneal concentrations > 1.2 μg/mL. Although there are no established breakpoints for cattle for perioperative/IP administration of ampicillin in the US, the Clinical Laboratory Standards Institute has approved breakpoints for cattle that could be extrapolated. The ampicillin‐susceptible breakpoint for cattle with metritis following IM ampicillin trihydrate at 11 mg/kg IM is 0.12 μg/mL for Enterobacterales (“CLSI VET01S‐ED7:2024” 2024) and showed IP administration maintained concentrations in plasma and peritoneal fluid longer than IM administration. A limitation of our data is that interpretive criteria for these bacteria from cattle have not been established, and the authors advise caution when generalizing the predicted MIC data across animal species to a particular antimicrobial drug, as it may result in inaccurate clinical outcome predictions (“CLSI VET01S‐ED7:2024” 2024).

While only mild changes within the abdominal cavity were observed following IP administration of ampicillin in these steers, these findings do not confirm the safety of IP infusion. In addition, there are no antibiotics labeled for cattle for surgical prophylaxis that are marketed in the United States, making the use of ampicillin IM or IP for this indication extralabel. Under all provisions outlined by the Animal Medicinal Drug Use Clarification Act, the use of ampicillin described in this study constitutes extralabel drug use, and practitioners should contact the Food Animal Residue Avoidance Databank (FARAD.org) for advice regarding meat withdrawal times following extralabel use of ampicillin trihydrate.

## 5 Conclusion

The use of intraoperative IP antibiotics in cattle as a rational antimicrobial therapy is controversial. Based on the results of the present study, ampicillin trihydrate administered once by the IP route at a dose of 11 mg/kg of body weight achieved higher concentrations for a short duration in the plasma and peritoneal fluid as compared to IM administration in cattle undergoing abdominal surgery. However, significant variation of pharmacological parameters associated with the IP route was noted in healthy cattle within the peritoneal cavity. Further work is needed to characterize the activity of ampicillin against common pathogens isolated from the incision and abdominal cavity of cattle undergoing abdominal surgery and establish susceptibility breakpoints to better guide practitioners on rational and judicious antimicrobial therapies.

## Author Contributions

**Danielle A. Mzyk:** study design, data acquisition, data analysis, drafting manuscript, critical review and editing of the manuscript. **Jennifer L. Halleran:** study design, data acquisition, critical review and editing of the manuscript. **Laura M. Neumann:** data acquisition, critical review and editing of the manuscript. **Ronald E. Baynes:** study design, critical review and editing of the manuscript. **Derek M. Foster:** study design, funding acquisition, critical review and editing of the manuscript. All authors have read and approved the manuscript.

## Ethics Statement

The authors confirm that the ethical policies of the journal, as noted on the journal's author guidelines page, have been adhered to and the appropriate ethical review committee approval has been received. The animal study was approved by the North Carolina State University Institutional Animal Care and Use Committee. The authors confirm that they have adhered to United States standards for the protection of animals used for scientific purposes.

## Conflicts of Interest

The authors declare no conflicts of interest.

## Supporting information


**Appendix S1:** jvp70023‐sup‐0001‐AppendixS1.docx.

## Data Availability

The data that support the findings of this study are available from the corresponding author upon reasonable request.
