# Supplementary material for: Pharmacokinetics of Ampicillin Trihydrate in Plasma, Interstitial, and Peritoneal Fluid Following Intraperitoneal or Intramuscular Administration in Steers at the Beginning of a Standing Flank Laparotomy
Source: J Vet Pharmacol Ther. 2025 Sep 4;49(1):44–54. doi: 10.1111/jvp.70023 (PMC12796777; doi:10.1111/jvp.70023)
Supplement: Supplementary file 1 — Appendix S1: jvp70023‐sup‐0001‐AppendixS1.docx. [file JVP-49-44-s001.docx]

**SUPPLEMENTAL MATERIAL**

*Range, LOD, LOQ, Intra- and Interday precision and accuracy for bovine plasma*

The concentration range for ampicillin in bovine plasma was 0.005 to 1.00 µg/ml with a correlation coefficient, R^2^, of 0.99 or higher. The limit of detection (LOD) was 0.001 µg/ml. The limit of quantification (LOQ) was 0.005 µg/ml. The intra- and interday precision and accuracy for cow plasma are shown in tables S1 and S2 below. Intraday precision and accuracy were determined by measuring five replicates (n=5) of four concentrations on the same day. Interday precision and accuracy was determined by measuring seven concentrations on eight (n=8) different days. Within the concentration range, the average accuracy ranges were 93.1% - 105.1% for intraday and 95.7% - 101.3% for interday. The precision (RSD%) ranges were 2.7%-13.7% for intraday and 8.4%-14.3% for interday.

*Table S1. Intraday precision and accuracy for bovine plasma*

| Spiked concentration (µg/mL) | Average Ampicillin concentration (n=5) | Standard deviation (µg/mL) | Relative Standard Deviation (RSD%) | Average Accuracy (%) |
| --- | --- | --- | --- | --- |
| 0.001 | 0.0007 | 0.0003 | 34.3 | 73.1 |
| 0.005 | 0.0048 | 0.0003 | 5.7 | 95.7 |
| 0.010 | 0.0093 | 0.0013 | 13.7 | 93.1 |
| 0.100 | 0.1051 | 0.0028 | 2.7 | 105.1 |

*Table S2. Interday precision and accuracy for bovine plasma*

| Spiked concentration (µg/mL) | Average Ampicillin concentration (n=8) | Standard deviation (µg/mL) | Relative Standard Deviation (RSD%) | Average Accuracy (%) |
| --- | --- | --- | --- | --- |
| 0.001 | 0.0010 | 0.0003 | 28.1 | 101.6 |
| 0.005 | 0.0050 | 0.0006 | 12.3 | 99.8 |
| 0.010 | 0.0101 | 0.0012 | 11.6 | 101.3 |
| 0.050 | 0.0505 | 0.0069 | 13.6 | 101.0 |
| 0.100 | 0.0979 | 0.0140 | 14.3 | 98.0 |
| 0.500 | 0.4909 | 0.0415 | 8.4 | 98.2 |
| 1.000 | 0.9576 | 0.0807 | 8.4 | 95.7 |

*Range, LOD, LOQ, Intra- and Interday precision and accuracy for cow ISF*

The concentration range for ampicillin in cow ISF was 0.0025 to 10 µg/mL with a correlation coefficient, R^2^, of 0.99 or higher. The limit of detection (LOD) was 0.0005 µg/mL. The limit of quantification (LOQ) was 0.001 µg/mL. The intra- and interday precision and accuracy for cow ISF are shown in Tables S3 and S4 below. Intraday precision and accuracy were determined by measuring four replicates (n=4) of four concentrations on the same day. Interday precision and accuracy was determined by measuring eight concentrations on five (n=5) different days. Within the concentration range, the average accuracy ranges were 100.7% - 101.9% for intraday and 80.0% - 112.2% for interday. The precision (RSD%) ranges were 2.7%-3.1% for intraday and 4.2%-12.9% for interday.

*Table S3. Intraday precision and accuracy for bovine ISF*

| Spiked concentration (µg/mL) | Average Ampicillin concentration (n=4) | Standard deviation (µg/mL) | Relative Standard Deviation (RSD%) | Average Accuracy (%) |
| --- | --- | --- | --- | --- |
| 0.00025 | 0.00021 | 0.00003 | 13.6 | 82.3 |
| 0.0005 | 0.00047 | 0.00003 | 6.3 | 93.2 |
| 0.001 | 0.0011 | 0.00003 | 3.1 | 100.7 |
| 0.05 | 0.051 | 0.014 | 2.7 | 101.9 |

*Table S4. Interday precision and accuracy for cow ISF*

| Spiked concentration (µg/mL) | Average Ampicillin concentration (n=5) | Standard deviation (µg/mL) | Relative Standard Deviation (RSD%) | Average Accuracy (%) |
| --- | --- | --- | --- | --- |
| 0.0005 | 0.0004 | 0.0001 | 23.1 | 74.1 |
| 0.001 | 0.009 | 0.001 | 6.9 | 91.4 |
| 0.01 | 0.011 | 0.005 | 4.3 | 111.8 |
| 0.10 | 0.11 | 0.0059 | 5.3 | 112.2 |
| 0.50 | 0.53 | 0.023 | 4.2 | 106.5 |
| 1.0 | 1.06 | 0.073 | 6.9 | 106.1 |
| 5.0 | 4.55 | 0.422 | 9.3 | 91.1 |
| 10.0 | 8.003 | 1.03 | 12.9 | 80.0 |
